# Safety and effectiveness of kidney transplantation using a donation after brain death donor with acute kidney injury: a retrospective cohort study

**DOI:** 10.1038/s41598-021-84977-1

**Published:** 2021-03-10

**Authors:** Kyeong Deok Kim, Kyo Won Lee, Sang Jin Kim, Okjoo Lee, Manuel Lim, Eun Sung Jeong, Jieun Kwon, Jaehun Yang, Jongwook Oh, Jae Berm Park

**Affiliations:** 1 Department of Surgery, Samsung Medical Center, Sungkyunkwan University School of Medicine, Seoul, 06355 Republic of Korea; 2 Department of Surgery, Samsung Changwon Hospital, Sungkyunkwan University School of Medicine, Changwon, Republic of Korea

**Keywords:** Kidney, Acute kidney injury

## Abstract

The use of kidneys from donation after brain death (DBD) donors with acute kidney injury (AKI) is a strategy to expand the donor pool. The aim of this study was to evaluate how kidney transplantation (KT) from a donor with AKI affects long-term graft survival in various situations. All patients who underwent KT from DBD donors between June 2003 and April 2016 were retrospectively reviewed. The KDIGO (Kidney Disease: Improving Global Outcomes) criteria were used to classify donor AKI. The cohort included 376 donors (no AKI group, n = 117 [31.1%]; AKI group n = 259 [68.9%]). Death-censored graft survival was similar according to the presence of AKI, AKI severity, and the AKI trend (*p* = 0*.*929, *p* = 0.077, and *p* = 0.658, respectively). Patients whose donors had AKI who received using low dose (1.5 mg/kg for three days) rabbit anti-thymocyte globulin (r-ATG) as the induction agent had significantly superior death-censored graft survival compared with patients in that group who received basiliximab (*p* = 0.039). AKI in DBD donors did not affect long-term death-censored graft survival. Low-dose r-ATG may be considered as an induction immunosuppression in recipients receiving kidneys with AKI because it showed better graft survival than basiliximab.

## Introduction

Kidney transplantation is the optimal choice for treating end-stage renal disease (ESRD) because it improves prognosis and quality of life compared with dialysis^1^. However, there is a huge gap between the demand for and supply of donor organs. The Korea Network for Organ Sharing (KONOS) reported that 2293 patients underwent kidney transplantation (KT) during 2019 in Korea, with one third of the recipients receiving grafts from donation after brain death (DBD) donors; 23,427 patients were still waiting for suitable organs in May 2019^2^.

In an attempt to increase the donor pool for kidney transplantation, the American United Network for Organ Sharing suggested the use of expanded criteria donors (ECDs) in 2002, even though those donors have 1.7-fold higher graft failure compared with standard criteria donors (SCDs)^3^. However, some recent studies reported comparable long-term graft survival in recipients who received kidneys from ECDs and SCDs, contrary to expectations^4,5^.

The use of kidneys from DBD donors who experienced acute kidney injury (AKI) at the time of death is another strategy to expand the donor pool^6^. Kwon et al.^7^ reported that delayed graft function (DGF) occurred more often when recipients received a kidney from a donor with AKI than from one without AKI. In addition, the rate of DGF tended to increase with the AKI stage^8,9^. Domagala et al.^10^ reported finding no difference in the rate of biopsy-proven acute rejection (BPAR) episodes between donors with and without AKI in the first year post-transplantation. Those reports thus found no relationship between donors with AKI and graft survival^7–10^. Nonetheless, debate continues regarding the use of donors with AKI. Several studies reported a significant difference in graft survival when using donors with AKI^11,12^. In addition, Yu et al.^13^ reported that the AKI trend is associated with graft survival rather than AKI severity. Park et al. reported that DBD donor KT recipients who received kidneys from ECDs with AKI showed worse long-term graft survival than other KT recipients^14^.

Therefore, in this study, we evaluated the effects of donor AKI on long-term graft survival after KT in various situations. In addition, we compared clinical outcomes, including the DGF rate, estimated glomerular filtration rate (eGFR), and rejection rate.

## Results

### Donor and recipient characteristics by donor AKI stage

117 (31.1%, 117/376) were included in the no AKI group, and 259 donors (68.9%, 259/376) were included in the AKI group. 102 (27.1%, 102/376), 71 (18.9%, 71/376), and 86 (22.9%, 86/376) patients had an AKI classified as KDIGO stage 1, 2, and 3, respectively. The KDIGO stage 3 group included 35 (40.7%, 35/86) donors who received renal replacement therapy before procurement. The comparison of donor characteristics according to the presence of AKI and AKI stage is shown in Table 1. The donor’s sex, history of DM and HCV, the proportion of AKI trend, and cold ischemia time did not differ significantly between groups. Donor age was higher in the AKI group (*p* = 0.025); in particular, the stage 1 AKI group had significantly older donors than the other two groups (*p* = 0.002). The history of HTN and proportion of ECDs did not differ significantly between the No AKI and AKI groups. However, donors with stage 1 AKI were more likely than other AKI donors to have HTN and be ECDs (*p* = 0.018). BMI differed significantly between the groups (*p* = 0.001), increasing with AKI stage. A cerebrovascular accident (CVA) caused brain death in the majority of the AKI group (*p* = 0.013), with stage 1 and 2 AKI having a higher proportion of CVA than the other groups (*p* = 0.011). KDRI and KDPI were significantly higher in the AKI group, with stage 1 AKI having significantly higher values than the other groups (*p* < 0.001). In addition, donor creatinine level (*p* < 0.001) was significantly higher in the AKI group (*p* < 0.001), and increased with AKI stage.

**Table 1:** Clinical characteristics of donors according to AKI stage.

	No AKI (n = 117)	AKI (n = 259)	*p-*value	KDIGO stage 1 (n = 102)	KDIGO stage 2 (n = 71)	KDIGO stage 3 (n = 86)	*p-*value
Age (years)	44.2 ± 16.0	47.9 ± 14.1	0.025	51.8 ± 15.5	44.5 ± 13.6	46.1 ± 11.7	0.002
Sex (n, % male)	75 (64.1)	176 (68.0)	0.463	63 (61.8)	56 (78.9)	57 (66.3)	0.101
BMI (kg/m^2^)	22.5 ± 3.2	23.7 ± 3.4	0.002	23.3 ± 3.3	23.5 ± 3.8	24.4 ± 3.2	0.001
**Cormobidities**							
Diabetes mellitus (n, %)	8 (7.0)	31 (12.4)	0.124	16 (16.0)	5 (7.6)	10 (11.9)	0.147
Hypertension (n, %)	26 (22.8)	65 (26.1)	0.501	34 (34.0)	19 (28.8)	12 (14.5)	0.018
HCV (n, %)	2 (1.8)	2 (0.8)	0.587	0 (0)	2 (2.8)	0 (0)	0.160
**Cause of death (n, %)**			0.013				0.011
Cerebrovascular accident	54 (46.1)	130 (50.2)		57 (55.9)	37 (52.1)	36 (41.9)	
Trauma	43 (36.8)	57 (22.0)		18 (17.6)	17 (30.0)	22 (25.6)	
Hypoxic brain damage	16 (13.7)	60 (23.2)		19 (18.6)	15 (21.1)	26 (30.2)	
Other	4 (3.4)	12 (4.6)		8 (7.9)	2 (2.8)	2 (2.3)	
Donor's status (ECD) (n, %)	28 (23.9)	83 (32.0)	0.110	42 (41.2)	16 (22.5)	25(29.1)	0.018
Kidney Donor Risk Index	1.08 ± 0.41	1.26 ± 0.45	0.001	1.38 ± 0.57	1.16 ± 0.34	1.18 ± 0.34	< 0.001
Kidney Donor Profile Index	50.4 ± 27.8	63.0 ± 23.9	< 0.001	67.1 ± 27.0	59.4 ± 21.3	61.2 ± 21.2	< 0.001
**Creatinine level (mg/dl)**							
Initial	0.99 ± 0.29	1.48 ± 1.15	< 0.001	1.17 ± 0.42	1.35 ± 0.75	1.95 ± 1.73	< 0.001
Peak	1.06 ± 0.29	2.60 ± 1.55	< 0.001	1.53 ± 0.40	2.25 ± 0.78	4.12 ± 1.64	< 0.001
Terminal	0.91 ± 0.27	2.00 ± 1.21	< 0.001	1.26 ± 0.43	1.72 ± 0.77	3.13 ± 1.33	< 0.001
**AKI trend (n, %)**			NA				0.299
Worsening	NA	73 (32.6)		32 (31.4)	20 (28.2)	21 (41.2)	
Improving	NA	151 (67.4)		70 (68.6)	51 (71.8)	30 (58.8)	
Cold ischemia time (min)	291.5 ± 170.7	280.3 ± 137.1	0.539	295.6 ± 149.8	277.4 ± 123.7	264.8 ± 131.5	0.478

Continuous variables given as mean ± SD.

*AKI* acute kidney injury, *KDIGO* kidney disease: improving global outcomes, *BMI* body mass index, *HCV* hepatitis c virus, *ECD* expanded criteria donor, *NA* not available.

Basiliximab was used more in recipients from the No AKI group, and low dose r-ATG was used more in recipients from the AKI group (*p* < 0.001); in particular, low dose r-ATG was used much more than basiliximab in recipients with stage 3 donors. The combination of tacrolimus, MMF, and MPD was used more in recipients from the AKI group for maintenance immunosuppression (*p* = 0.006) (Table 2).

**Table 2:** Clinical characteristics of recipient according to AKI stage.

	No AKI (n = 117)	AKI (n = 259)	*p-*value	KDIGO stage 1 (n = 102)	KDIGO stage 2 (n = 71)	KDIGO stage 3 (n = 86)	*p-*value
Age (years)	46.2 ± 11.9	48.2 ± 11.9	0.119	48.2 ± 10.7	47.9 ± 11.3	48.3 ± 11.0	0.481
Sex (n, % male)	63 (53.8)	159 (61.4)	0.168	57 (55.9)	50 (70.4)	52 (60.5)	0.132
BMI (kg/m^2^)	23.6 ± 3.5	23.2 ± 3.4	0.313	23.2 ± 3.6	22.6 ± 2.9	23.8 ± 3.4	0.118
**Cormobidities**							
Diabetes mellitus (n, %)	24 (20.5)	52 (20.1)	0.922	25 (24.5)	9 (12.7)	18 (20.9)	0.294
Hypertension (n, %)	95 (81.2)	211 (81.5)	0.950	82 (80.4)	61 (85.9)	68 (79.1)	0.720
**Cause of ESRD (n, %)**			0.739				0.524
DM nephropathy	23 (19.7)	46 (17.8)		21 (20.6)	9 (12.7)	16 (18.6)	
Hypertension	14 (12.0)	42 (16.2)		22 (21.6)	10 (14.1)	10 (11.6)	
GN	28 (23.9)	64 (24.7)		26 (25.5)	16 (22.5)	22 (25.6)	
ADPKD	7 (6.0)	10 (3.9)		4 (3.9)	3 (4.2)	3 (3.5)	
Other	45 (38.5)	97 (37.5)		29 (28.4)	33 (46.5)	35 (40.7)	
Modality of dailysis (n, % HD)	95 (81.2)	202 (78.0)	0.371	80 (78.4)	55 (77.5)	67 (77.9)	0.685
Duration of dailysis (years)	6.2 ± 3.7	6.3 ± 3.6	0.797	5.9 ± 3.5	6.5 ± 3.4	6.6 ± 3.9	0.582
cPRA ≥ 50% (n, %)	5 (4.4)	17 (6.8)	0.375	7 (7.2)	4 (5.9)	6 (7.1)	0.818
**HLA mismatch**							
HLA class I	1.97 ± 1.34	2.28 ± 1.19	0.024	2.23 ± 1.22	2.21 ± 1.30	2.40 ± 1.06	0.098
HLA class II	0.86 ± 0.72	0.91 ± 0.63	0.568	0.86 ± 0.68	0.82 ± 0.54	1.03 ± 0.62	0.104
Donor specific antigen (n, %)	4 (3.5)	6 (2.5)	0.732	3 (3.2)	1 (1.5)	2 (2.4)	0.920
**Induction immunosuppression (n, %)**			< 0.001				< 0.001
Basiliximab	82 (70.1)	110 (42.5)		53 (52.0)	39 (55.0)	18 (20.9)	
High dose r-ATG	22 (18.8)	59 (22.8)		21 (20.5)	16 (22.5)	22 (25.6)	
Low dose r-ATG	13 (11.1)	90 (34.7)		28 (27.5)	16 (22.5)	46 (53.5)	
**Maintenance immunosuppression (n, %)**			0.006				0.042
Cyclosporine + MMF + MPD	22 (18.8)	23 (8.9)		9 (8.8)	8 (11.3)	6 (7.0)	
Tacrolimus + MMF + MPD	95 (81.2)	236 (91.1)		93 (91.2)	63 (88.7)	80 (93.0)	

Continuous variables given as mean ± SD.

*AKI* acute kidney injury, *KDIGO* kidney disease: improving global outcomes, *BMI* body mass index, *ESRD* end stage renal disease, *DM* diabetes mellitus, *ADPKD* autosomal dominant polycystic kidney disease, *HD* hemodialysis, *cPRA* calculated panel reactive antibody, *HLA* human leukocyte antigen, *ATG* anti-thymocyte globulin, *MMF* mycophenolate mofetil, *MPD* methylprednisolone.

### Death-censored graft survival according to various conditions

**Figure 1:**
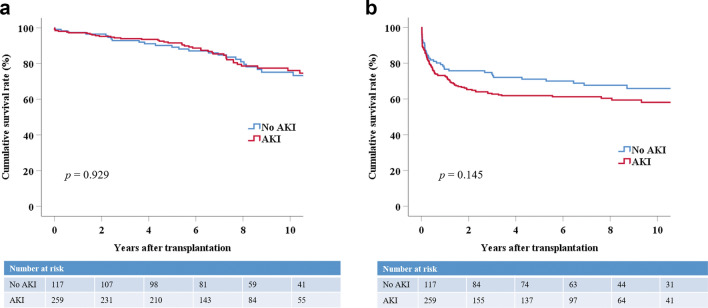
Death-censored and rejection-free graft survival between the No AKI and AKI groups. (**a**) Death-censored graft survival between the No AKI and AKI groups. Group comparisons were performed using the Kaplan–Meier and log-rank tests. (**b**) Rejection-free graft survival between the No AKI and AKI groups. Group comparisons were performed using the Kaplan–Meier and log-rank tests. *AKI* acute kidney injury.

**Figure 2:**
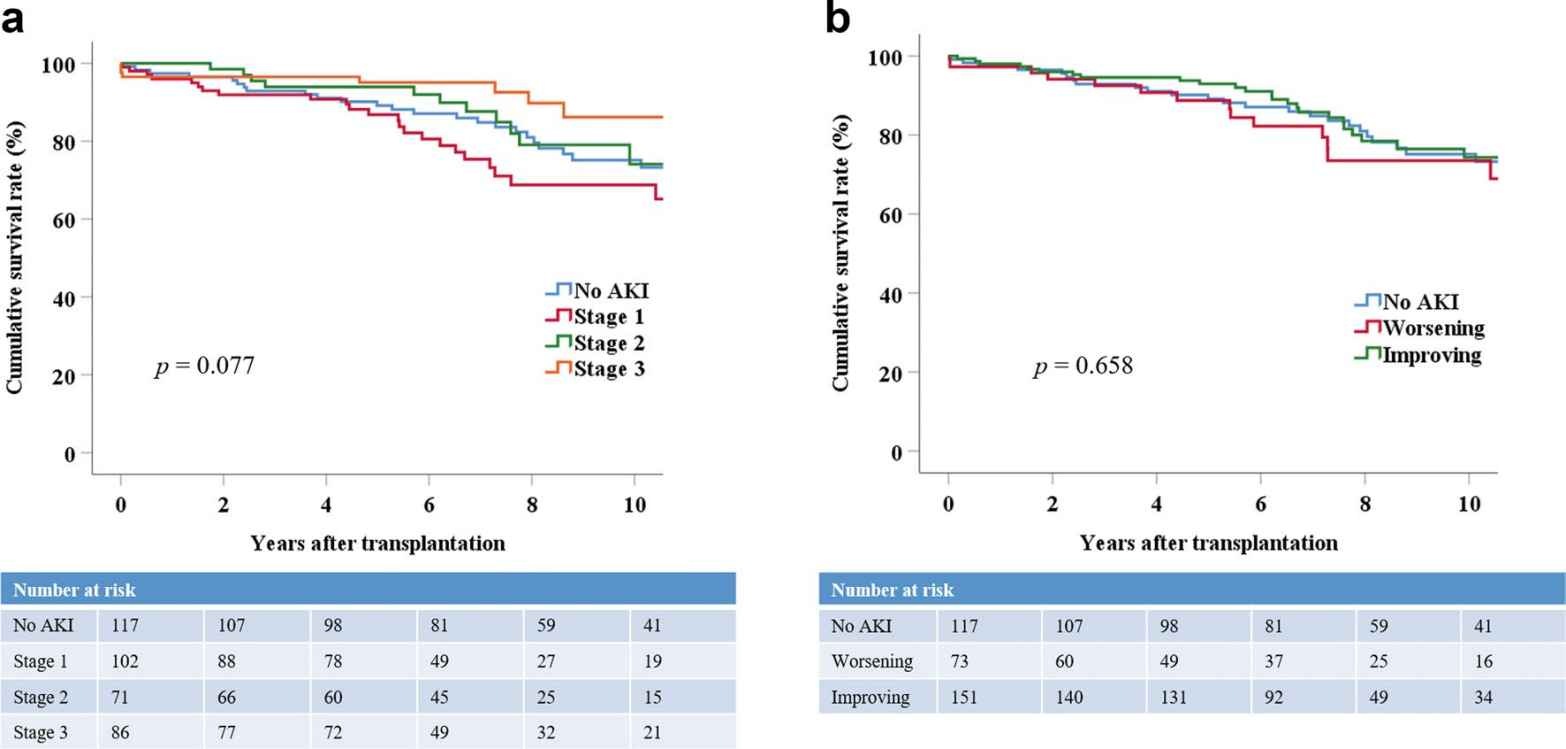
Death-censored graft survival stratified by AKI severity and trend. (**a**) Death-censored graft survival stratified by AKI severity. Group comparisons were performed using the Kaplan–Meier and log-rank tests. (**b**) Death-censored graft survival stratified by the AKI trend. Group comparisons were performed using the Kaplan–Meier and log-rank tests.

Donor AKI itself did not affect death-censored graft survival; the 1-, 5-, and 10-year death-censored graft survival rates were 97.4%, 89.2%, and 75.1%, respectively, in recipients in the No AKI group and 97.3%, 91.6%, and 76.1%, respectively, in recipients in the AKI group (*p* = 0.929) (Fig. 1a). This trend was also observed when the AKI group was stratified by KDIGO stage. The KDIGO stage 3 group (the most severe AKI group) did not show the worst graft survival. The 1-, 5-, and 10-year death-censored graft survival rates were similar among the KDIGO groups: Stage 1 (96.0%, 86.8%, and 68.8%), stage 2 (100%, 93.9%, and 74.1%), and stage 3 (96.5%, 95.1%, and 86.2%, *p* = 0.077) (Fig. 2a). The AKI trends did not influence death-censored graft survival either. The 1-, 5-, and 10-year death-censored graft survival rates were as follows: No AKI (97.4%, 89.2%, and 75.1%), worsening AKI (97.3%, 88.7%, and 73.5%), and improving AKI (98.0%, 93.0%, and 74.3%, *p* = 0.658) (Fig. 2b).

**Figure 3:**
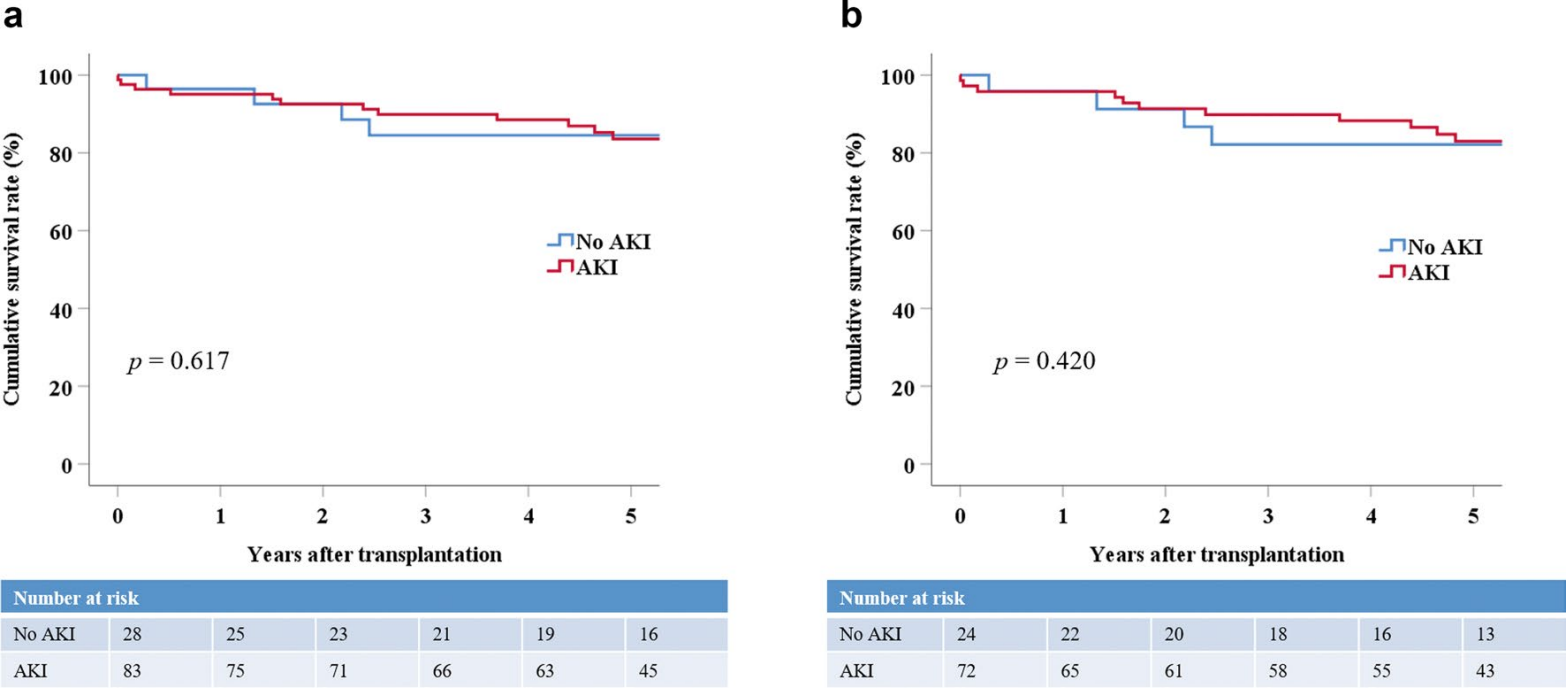
Death-censored graft survival in recipients receiving kidneys from ECD and high KDPI (≥ 80) donors stratified by the presence of AKI. (**a**) Death-censored graft survival in recipients receiving kidneys from ECDs stratified by the presence of AKI. Group comparisons were performed using the Kaplan–Meier and log-rank tests. (**b**) Death-censored graft survival in recipients receiving kidneys from high KDPI (≥ 80) donors stratified by the presence of AKI. Group comparisons were performed using the Kaplan–Meier and log-rank tests. *ECD* expanded criteria donor, *KDPI* kidney donor profile index.

Donor AKI did not have a negative effect on death-censored graft survival in the ECD KT group. The 1- and 5-year death-censored graft survival rates in the ECD KT group did not differ significantly between the No AKI (96.4% and 84.5%) and AKI groups (95.1% and 83.6%, *p* = 0.617) (Fig. 3a). AKI also did not negatively affect death-censored graft survival in the high KDPI (≥ 80) group. The 1- and 5-year death-censored graft survival rates in the high KDPI (≥ 80) group did not differ significantly between the No AKI (95.8% and 82.1%) and AKI groups (95.8% and 83.0%, *p* = 0.420) (Fig. 3b).

### Other clinical outcomes

The DGF rate was significantly higher in recipients with donors in the AKI group (*p* < 0.001) and tended to increase with the AKI stage (5.1%, 17.6%, 15.5%, and 61.6%, for the No AKI and stage 1, 2, and 3 AKI groups, respectively *p* < 0.001) (Table 3). However, the DGF rate did not have a negative effect on death-censored graft survival in the univariate analysis (*p* = 0.126) (Table 4).

**Table 3:** Clinical outcomes.

	No AKI (n = 117)	AKI (n = 259)	*p-*value	KDIGO stage 1 (n = 102)	KDIGO stage 2 (n = 71)	KDIGO stage 3 (n = 86)	*p-*value
DGF (n, %)	6 (5.1)	82 (31.7)	< 0.001	18 (17.6)	11 (15.5)	53 (61.6)	< 0.001
Graft failure (n, %)	26 (22.2)	45 (17.4)	0.266	24 (23.5)	12 (16.9)	9 (10.5)	0.092
Nadir SCr (< 3 month)	1.18 ± 0.38	1.26 ± 0.38	0.049	1.26 ± 0.45	1.26 ± 0.34	1.28 ± 0.33	0.265
Time to nadir SCr (days)	56.6 ± 25.2	57.8 ± 24.9	0.661	53.5 ± 24.6	59.6 ± 25.3	61.5 ± 24.6	0.160
2-year SCr	1.25 ± 0.46	1.29 ± 0.38	0.417	1.30 ± 0.42	1.34 ± 0.40	1.25 ± 0.32	0.511
4-year SCr	1.28 ± 0.59	1.37 ± 0.62	0.205	1.43 ± 0.75	1.38 ± 0.53	1.29 ± 0.53	0.313
6-year SCr	1.18 ± 0.36	1.47 ± 0.79	< 0.001	1.54 ± 0.96	1.58 ± 0.89	1.30 ± 0.45	0.004
8-year SCr	1.16 ± 0.39	1.35 ± 0.53	0.027	1.29 ± 0.49	1.40 ± 0.58	1.36 ± 0.53	0.138
10-year SCr	1.20 ± 0.44	1.35 ± 0.71	0.208	1.46 ± 1.00	1.26 ± 0.40	1.33 ± 0.54	0.476
2-year eGFR, mL/min/1.73m^2^	59.9 ± 17.9	57.5 ± 16.1	0.214	56.9 ± 17.2	57.3 ± 15.9	58.2 ± 15.1	0.616
4-year eGFR, mL/min/1.73m^2^	59.3 ± 18.8	56.7 ± 19.4	0.259	54.4 ± 17.9	56.9 ± 17.5	59.0 ± 22.2	0.320
6-year eGFR, mL/min/1.73m^2^	60.5 ± 18.4	53.8 ± 20.4	0.014	52.8 ± 22.5	53.1 ± 21.3	55.5 ± 17.5	0.083
8-year eGFR, mL/min/1.73m^2^	62.7 ± 21.8	57.0 ± 21.8	0.144	58.2 ± 20.2	56.9 ± 18.9	55.9 ± 26.0	0.517
10-year eGFR, mL/min/1.73m^2^	60.8 ± 19.4	58.0 ± 20.9	0.501	57.5 ± 23.6	61.3 ± 18.6	56.1 ± 20.7	0.796
Rejection rate (n, %)	37 (31.6)	98 (37.8)	0.245	43 (42.2)	33 (46.5)	22 (25.6)	0.018
ACR	32 (27.4)	92 (35.5)	0.119	40 (39.2)	31 (43.7)	21 (24.4)	0.019
AMR	3 (2.6)	15 (5.8)	0.175	4 (3.9)	6 (8.5)	5 (5.8)	0.293
Combined ACR + AMR	6 (5.1)	0 (0)	0.001	0 (0)	0 (0)	0 (0)	0.004
Follow up duration (years)	8.0 [5.3–11.0]	6.6 [4.3–8.7]	0.002	5.9 [4.1–8.2]	7.0 [4.9–9.1]	6.8 [4.8–9.7]	0.003

Continuous variables given as mean ± SD or as median [P25-P75].

*AKI* acute kidney injury, *KDIGO* kidney disease: improving global outcomes, *DGF* delayed graft function, *SCr* serum creatinine, *eGFR* estimated glomerular filtration rate, *ACR* acute cellular rejection, *AMR* antibody mediated rejection.

**Table 4:** Univariate and multivariate analyses of risk factors for graft failure.

Variables	Univariate		Multivariate	
	HR (95% CI)	*p-*value	HR (95% CI)	*p-*value
Recipient age	1.011 (0.988–1.033)	0.348	1.010 (0.984–1.036)	0.463
Male recipient	1.406 (0.867–2.281)	0.165	1.439 (0.821–2.521)	0.203
Recipient BMI	1.016 (0.950–1.087)	0.644		
HLA class I mismatch	1.048 (0.865–1.269)	0.634		
HLA class II mismatch	1.127 (0.791–1.606)	0.508		
Male donor	0.776 (0.479–1.258)	0.304		
Donor BMI	1.033 (0.965–1.106)	0.345		
Terminal serum Cr	0.821 (0.634–1.065)	0.137		
KDRI	3.000 (1.906–4.722)	< 0.001	5.202 (2.959–9.145)	< 0.001
**KDIGO stage**		0.088		
No AKI	1 (Ref.)			
Stage 1	1.460 (0.835–2.553)	0.184		
Stage 2	0.905 (0.456–1.797)	0.776		
Stage 3	0.556 (0.260–1.188)	0.130		
AKI	0.978 (0.602–1.589)	0.929	0.637 (0.371–1.096)	0.103
**Trend**		0.659		
No AKI	1 (Ref.)			
Worsening	1.234 (0.653–2.333)	0.518		
Improving	0.919 (0.533–1.586)	0.762		
DGF	1.522 (0.888–2.607)	0.126	1.444 (0.683–3.052)	0.336
**Induction immunosuppression**		0.012		< 0.001
Basiliximab	1 (Ref.)		1 (Ref.)	
High dose r-ATG	1.813 (1.107–2.971)	0.018	1.752 (1.027–2.988)	0.040
Low dose r-ATG	0.589 (0.244–1.422)	0.239	0.187 (0.061–0.574)	0.003
**Maintenance immunosuppression**		0.051		0.097
Cyclosporine + MMF + MPD	1 (Ref.)		1 (Ref.)	
Tacrolimus + MMF + MPD	0.581 (0.337–1.002)		0.600 (0.328–1.097)	
Nadir serum SCr in 3 months	2.535 (1.456–4.415)	0.001	1.272 (0.657–2.464)	0.476
Time to nadir serum SCr	1.003 (0.993–1.013)	0.551		
Rejection episode	2.303 (1.437–3.688)	0.001	3.016 (1.801–5.051)	< 0.001

*BMI* body mass index, *HLA* human leukocyte antigen, *Cr* creatinine, *KDIGO* kidney disease: improving global outcomes, *AKI* acute kidney injury, *DGF* delayed graft function, *ATG* anti-thymocyte globulin, *MMF* mycophenolate mofetil, *MPD* methylprednisolone.

**Figure 4:**
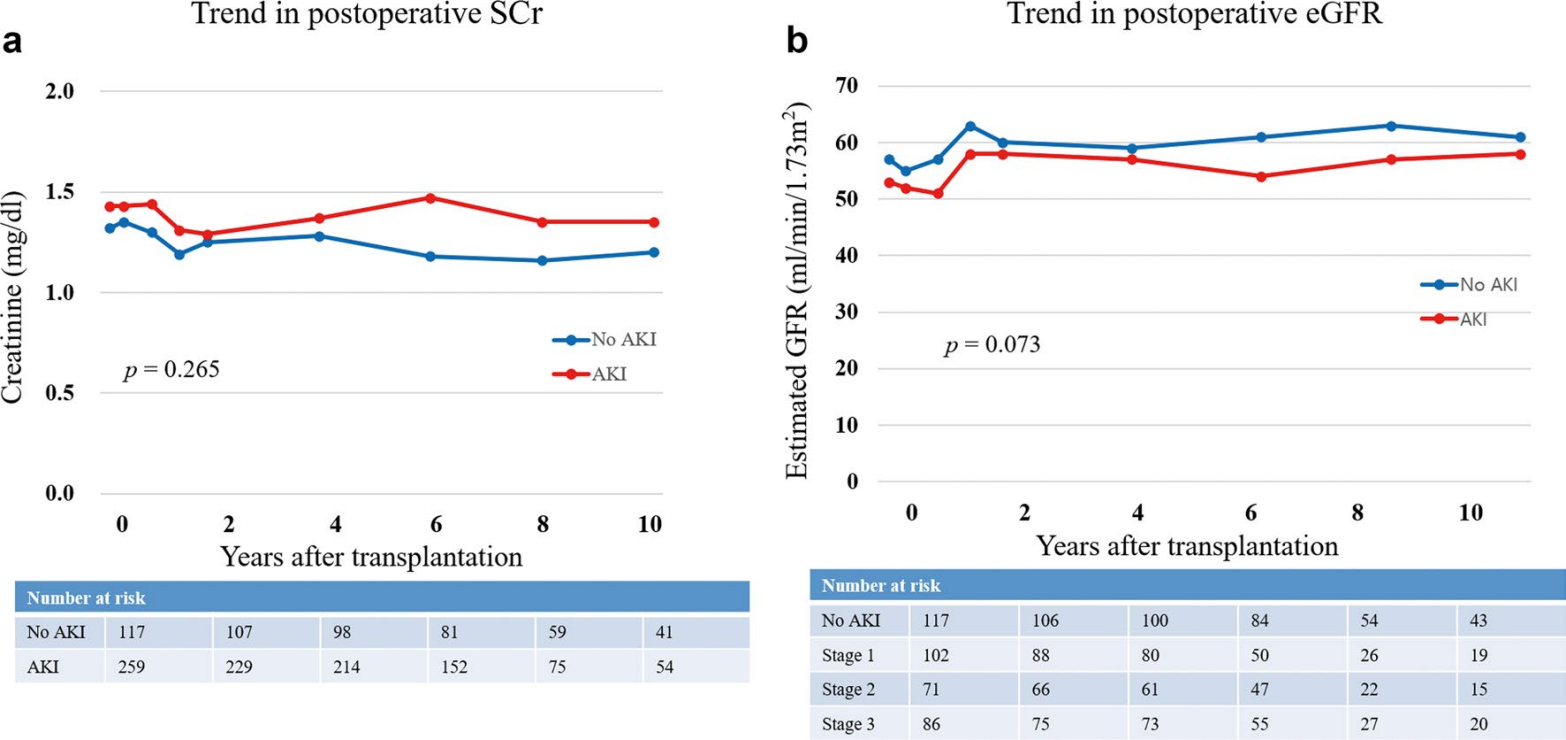
Trend in postoperative SCr and eGFR between the No AKI and AKI groups. (**a**) Trend in postoperative SCr stratified by the presence of AKI. Group comparisons were performed using a linear mixed model. (**b**) Trend in postoperative creatinine stratified by the presence of AKI. Group comparisons were performed using a linear mixed model. *SCr* serum creatinine, *eGFR* estimated glomerular filtration rate.

Nadir SCr within three months post-KT was significantly higher in the AKI group (*p* = 0.049). However, this value did not differ significantly based on AKI severity (*p* = 0.265). The nadir SCr seemed to affect death-censored graft survival in the univariate analysis (*p* = 0.001); however, it was not significant in the multivariate analysis (*p* = 0.476) (Table 4). The trend of kidney allograft function, estimated by SCr and eGFR, was similar between the No AKI and AKI groups (*p* = 0.265 and *p* = 0.073, respectively) (Fig. 4).

The rejection rate was similar between the No AKI and AKI groups (*p* = 0.245), but it was significantly higher in the groups with stage 1 and 2 AKI (*p* = 0.018). Acute cellular rejection (ACR) composed a large portion of the rejection cases. Rejection-free graft survival was similar between the No AKI and AKI groups (*p* = 0.145) (Fig. 1b). However, it was significantly different between No AKI and AKI groups (KDIGO stage 1 and 2) (*p* = 0.018) (Fig. S1).

### Univariate and multivariate risk factors for death-censored graft survival

The presence of donor AKI, AKI severity, and the AKI trend were not significant factors associated with death-censored graft survival. In the multivariate analysis, the use of high dose r-ATG as an induction agent, KDRI, and rejection episodes had a negative effect on death-censored graft survival (*p* = 0.040, < 0.001, and < 0.001). The use of low dose r-ATG as an induction agent had a positive effect on death-censored graft survival (*p* = 0.003) (Table 4).

In patients with AKI donors, the use of high dose r-ATG as an induction agent, KDRI, and rejection episodes had a negative effect on death-censored graft survival (*p* = 0.025, < 0.001, and 0.002). The use of low dose r-ATG as an induction agent had a positive effect on death-censored graft survival (*p* = 0.039) (Table 5).

**Table 5:** Univariate and multivariate analyses of risk factors for graft failure in patients with AKI donors.

Variables	Univariate		Multivariate	
	HR (95% CI)	*p-*value	HR (95% CI)	*p-*value
Recipient age	1.008 (0.978–1.038)	0.614	1.019 (0.983–1.056)	0.305
Male recipient	1.180 (0.641–2.174)	0.595	0.973 (0.424–2.233)	0.949
Recipient BMI	1.035 (0.954–1.122)	0.410		
HLA class I mismatch	0.981 (0.767–1.256)	0.881		
HLA class II mismatch	1.132 (0.708–1.808)	0.605		
Male donor	0.903 (0.485–1.679)	0.746		
Donor BMI	1.011 (0.929–1.100)	0.795		
Terminal serum Cr	0.787 (0.564–1.043)	0.091		
KDRI	2.737 (1.549–4.835)	0.001	4.247 (1.934–9.325)	< 0.001
**Trend**		0.353		0.988
Worsening	1 (Ref.)		1 (Ref.)	
Improving	0.740 (0.392–1.398)		0.995 (0.478–2.068)	
DGF	1.183 (0.628–2.227)	0.602		
**Induction immunosuppression**		0.013		0.001
Basiliximab	1 (Ref.)		1 (Ref.)	
High dose r-ATG	2.062 (1.097–3.878)	0.025	2.234 (1.108–4.503)	0.025
Low dose r-ATG	0.600 (0.219–1.650)	0.323	0.242 (0.063–0.932)	0.039
**Maintenance immunosuppression**		0.117		0.381
Cyclosporine + MMF + MPD	1 (Ref.)		1 (Ref.)	
Tacrolimus + MMF + MPD	0.563 (0.275–1.154)		0.692 (0.304–1.576)	
Nadir serum SCr in 3 months	2.535 (1.456–4.415)	0.062	1.627 (0.654–4.050)	0.295
Time to nadir serum SCr	1.008 (0.995–1.021)	0.224		
Rejection episode	1.874 (1.040–3.375)	0.036	2.976 (1.506–5.881)	0.002

*BMI* body mass index, *HLA* human leukocyte antigen, *Cr* creatinine, *DGF* delayed graft function, *ATG* anti-thymocyte globulin, *MMF* mycophenolate mofetil, *MPD* methylprednisolone.

## Discussion

In this study, we found that the DGF rate tended to increase with the donor AKI stage. However, AKI in DBD donors did not affect long-term allograft function. The presence of donor AKI, AKI severity, and the AKI trend did not affect death-censored graft survival. In addition, AKI in ECD or high KDPI donors did not change death-censored graft survival. In the AKI group, recipients treated with low dose r-ATG for induction immunosuppression showed better graft survival than patients treated with other types of induction immunosuppression in the multivariate analysis.

Previous studies showed that donors with AKI were associated with a higher rate of DGF^7,8,10,15^, and that the DGF rate was higher with AKIN stage 2 and 3 disease^9,16^. Similarly in our study, the DGF rate tended to increase with the donor AKI stage, especially in stage 3. Although some studies showed that DGF was associated with a greater risk of graft loss^17–19^, DGF did not affect long-term death-censored graft survival in our study. That might have been because we used more r-ATG for induction in the AKI group; previous studies reported that r-ATG could ameliorate ischemic reperfusion injury (IRI) and reduce the incidence of DGF^20,21^. DGF is a risk factor for the development of early acute rejection^17,22^. However, previous studies reported that the acute rejection rate was not significantly higher among patients whose donors had AKI than among other KT recipients^7,8,10,15^. We also observed that donor AKI did not affect the acute rejection rate or rejection-free survival. However, when we compared the recipients receiving No AKI kidneys and recipients receiving AKI kidneys of KDIGO stage 1 and 2 only, the rejection free graft survival was significantly superior in the former group. We speculate that this may be because twice as much of the recipients who received AKI kidneys of KDIGO stage 3 used low dose r-ATG (53.5%, n = 46) compared to those who received KDIGO stage 1 (27.5%, n = 28) or 2 kidneys (22.5%, n = 16).Many studies reported that DBD donors with AKI were not associated with graft failure^8–10,15,23,24^, although some studies have shown otherwise^11,12^. Our results show that long-term death-censored graft survival in patients with a DBD donor with AKI was not inferior to that in patients with a DBD donor without AKI. The 10-year death-censored graft survival was 76.1% in the AKI group and 75.1% in the No AKI group. In addition, long-term graft function and the eGFR trend were similar between the two groups.

Park et al.^14^ reported that the graft survival of kidneys from ECDs with AKI was significantly worse than that in other groups (ECDs without AKI, SCDs with AKI, and SCDs without AKI). On the other hand, Ko et al.^25^ found no significant difference in the graft survival rate among those four groups. In our cohort, long-term death-censored graft survival was similar between ECDs with and without AKI. Similarly, long-term death-censored graft survival did not differ significantly between high KDPI (≥ 80) donors with and without AKI.

Boffa et al.^11^ reported that the presence and severity of AKI, especially in kidneys from donors with AKIN stage 3 injury, led to inferior graft survival. Yu et al.^13^ reported that the AKI trend, specifically DBD donors with worsening AKI, led to inferior graft survival. However, we found that the AKI trend and severity did not affect death-censored graft survival. On the contrary, although this finding was not statistically significant, graft survival tended to be superior in recipients receiving KDIGO stage 3 kidneys than in recipients receiving No AKI, KDIGO stage 1 or 2 kidneys. In the case of KDIGO stage 1 kidneys, graft survival tended to be inferior because of more ECD and higher KDPI. However, ECD was similar in the rest of the groups except for KDIGO stage 1 kidneys, and even KDPI was lower in the No AKI group. Among the four groups, there was a significant difference in the choice of induction immunosuppressive agents. Compared with the other groups, more recipients receiving KDIGO stage 3 kidneys received low dose r-ATG for induction therapy. We speculate that the difference in induction agents might have affected graft survival.

In the multivariate analysis, KDRI and rejection episodes had a negative effect on death censored graft survival. the choice of immunosuppressive induction agent in patients with AKI donors significantly affected death-censored graft survival, which was significantly inferior when using high dose r-ATG as an induction immunosuppressive agent (HR 2.234, *p* = 0.025) and significantly superior when using low dose r-ATG (HR 0.242, *p* = 0.039), even though donor age and the proportions of ECD, KDRI, and KDPI donors were significantly higher in the low dose r-ATG group (*p* < 0.001) (Table S1). Lee et al.^26^ also reported that in patients who received kidneys from deceased donors with AKIN stage 1 or 2 AKI, graft survival was better when low dose r-ATG was used as induction therapy, although their finding was not statistically significant. IRI is a major cause of AKI in donors^27^, inducing leukocytes to adhere to the venular endothelium^28^. ATG suppresses immune responses after IRI by causing the apoptosis of T cells in peripheral lymphoid organs^29^, which prevents leukocyte clotting and capillary plugging and helps to preserve the microcirculation of the allograft^30^. Although excessive doses of ATG can worsen allograft survival, adequate doses of ATG appear to have a positive effect on allografts by reducing the consequences of AKI.

In Korea, only 10% of the patients waiting for suitable organs receive KT, and one third of the donors are DBD donors. Donation of patients deceased due to brain death has reduced since 2016. The proportion of donors of age older than 50 years has increased from 52.1% in 2014 to 61.5% in 2019^2^. Given the increased proportion of ECDs and high KDPI donors, our finding that donor AKI does not affect graft survival could decrease the number of discarded organs and increase the number of KT recipients.

This study has some limitations. This is a retrospective, single-center cohort study and the period of this cohort is 13 years which is quite long. During this period, selection biases were inevitable due to the evolution of donor selection criteria, changes in the immunosuppression protocol, and the accumulation of experience of clinicians. For this reason, the low dose r-ATG was used later than the high dose r-ATG. Thus, the follow-up period using the low dose r-ATG was shorter and the average age of the donor was higher. Also, the follow-up period was shorter in AKI group than No AKI group. Transplanted kidneys with AKI were clinically chosen instead of being discarded. Thus, we might have underestimated the effect of AKI by discarding kidneys due to poor expected outcomes. In addition, the AKI stages might be not correct because we used the KDIGO definition without considering urine output when classifying donor AKI stage.

In conclusion, AKI in DBD donors negatively affected the DGF rate. However, it did not affect long-term graft function or death-censored graft survival. The use of high dose-r-ATG as an induction agent, KDRI, and rejection episodes had a negative effect on death-censored graft survival. Low dose r-ATG may be considered as an induction immunosuppression in recipients receiving kidneys with AKI because it produced better graft survival than basiliximab.

## Materials and methods

### Ethical approval and informed consent

This retrospective study was approved by the Institutional Review Board of Samsung Medical Center (IRB No. 2020-03-170), and the need for informed consent was waived. All methods were carried out in accordance with Declaration of Helsinki.

### Patients and data

**Figure 5:**
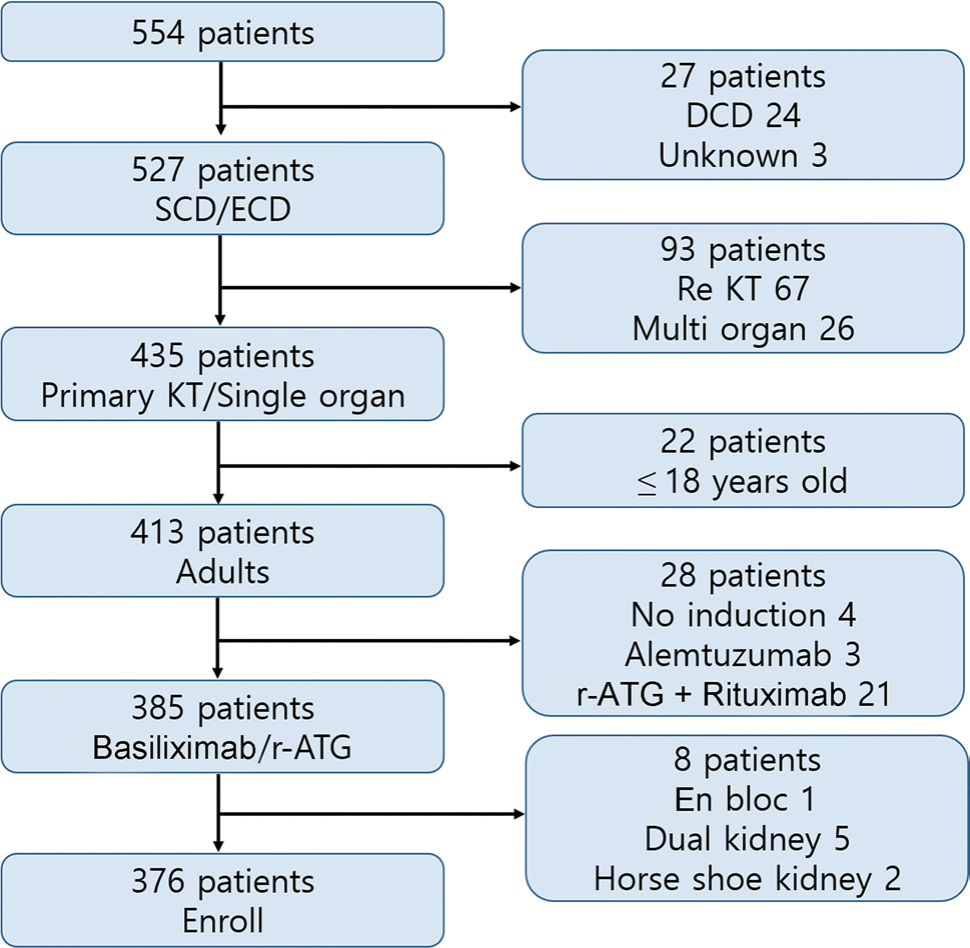
Flow chart of the patient selection process. *SCD* standard criteria donor, *ECD* expanded criteria donor, *DCD* donation after circulatory death, *KT* kidney transplantation, *r-ATG* rabbit anti-thymocyte globulin.

We retrospectively reviewed all patients who underwent KT from DBD donors at Samsung Medical Center in Seoul, Korea, between June 2003 and April 2016. Figure 5 shows a flow chart summarizing patient enrollment. We included 527 transplants from DBD donors without any cardiac death donors. Primary kidney transplantations and single organ transplantations are included. We excluded patients younger than 18 and those who did not receive immunosuppressive induction therapy, as well as those who received alemtuzumab or a combination of rituximab and rabbit anti-thymocyte globulin (r-ATG). En-bloc transplants, dual kidney transplants, and horseshoe kidney transplants were also excluded. In the end, 376 patients were enrolled in this study.

### Immunosuppression

The immunosuppressive induction agents used were basiliximab and high and low dose r-ATG. We usually use basiliximab for patients who receive SCD kidneys and r-ATG for patients who receive ECD kidneys. However, we gave induction agents considering individual situations, including immunologic risks and physical conditions. The recipients who received 20 mg of basiliximab as an induction agent were injected intravenously twice on the operative day and on postoperative day 4. We used high dose r-ATG (1.5 mg/kg for more than five days) before July 2011 and low dose r-ATG (1.5 mg/kg for 3 days) after that time. r-ATG was initiated on the operative day and administered daily. 500 mg of intravenous methylprednisolone (MPD) was also used for two days starting on the operative day and tapering as scheduled.

Maintenance immunosuppression was achieved with a triple immunosuppressive regimen consisting of calcineurin inhibitors (CNIs), mycophenolate mofetil (MMF) and MPD. Each patient received MMF and MPD and then either cyclosporine or tacrolimus.

### Clinical parameters and outcomes

We retrospectively analyzed the donor and recipient data. Donors were compared based on their age; sex; body mass index (BMI) (kg/m^2^); history of diabetes mellitus (DM), hypertension (HTN), and hepatitis C virus (HCV); cause of death; ECD status; Kidney Donor Risk Index (KDRI); Kidney Donor Profile Index (KDPI); and the initial, peak, and terminal serum creatinine (SCr) according to AKI stage. We calculated the KDRI and KDPI by using the Organ Procurement and Transplantation Network calculator^31^. We received all serial SCr values between the donor’s hospitalization and organ procurement from KONOS.

The recipient data collected were age, sex, BMI, cause of ESRD, modality and period of dialysis before KT, percentage of panel-reactive antibodies, number of human leukocyte antigen (HLA) mismatches, induction immunosuppressant type, and maintenance immunosuppression agents.

We used the KDIGO criteria to classify donor AKI stage because a previous report showed that the KDIGO criteria are more useful for predicting DGF in KT recipients than the AKIN (AKI Network) criteria^32^. According to the KDIGO criteria, stage 1 is any of the following: increase in SCr by ≥ 0.3 mg/dL within 48 h (h), increase in SCr to ≥ 1.5 times baseline that is known or presumed to have occurred within 7 days, or a reduction in urine output (< 0.5 mL/kg/h for 6 h). Stage 2 is an increase in SCr to 2.0–2.9 times baseline or a reduction in urine output to < 0.5 mL/kg/h for 12 h. Stage 3 is an increase in SCr to 3.0 times baseline or ≥ 4.0 mg/dL or receipt of renal replacement therapy (RRT); in patients < 18 years, it is defined as a decrease in eGFR to < 35 ml/min per 1.73 m^2^ or a reduction in urine output (< 0.3 mL/kg/h for 24 h or anuria for 12 h)^33^. We used the KDIGO criteria without urine output because that information was not consistently documented in the records for brain-dead donors from other hospitals. The AKI trend was defined using the differences between the peak and terminal SCr levels during the donor management period. When the terminal SCr was lower than the peak SCr, it was defined as improving AKI, and when the terminal SCr equaled the peak SCr, it was defined as worsening AKI^13^. However, donors who received renal replacement therapy were excluded from the AKI trend analysis. We used the Revised Bedside Schwartz Formula to estimate GFR for patients less than 18 years old and the Modification of Diet in Renal Disease equation to estimate GFR for patients over 18. BPAR was defined and classified according to the Banff 2013 classification.

Kidneys were discarded if they were grossly discolored or atrophied or if the donor's SCr had elevated for more than 7 days without renal replacement therapy.

The primary outcome was the effect of donor AKI, AKI severity, and the AKI trend on death-censored allograft survival, as well as the effect of AKI on death-censored graft survival for KTs using kidneys from ECDs and high KDPI donors. The secondary outcomes were the incidence of DGF, nadir SCr, time to nadir SCr over 3 months, changes in allograft function based on Cr and eGFR, and the rejection rate. We also investigated whether graft survival was influenced by the type of induction immunosuppression.

### Statistical analyses

Continuous variables are presented as means ± standard deviations and were compared using the student’s t-test and one-way analysis of variance or as medians with interquartile ranges, which were compared using the Kruskal–Wallis test. Categorical variables are presented as numbers and percentages and were compared by chi-square tests. Graft failure was defined as restarting dialysis or re-transplantation. The trends of postoperative SCr and eGFR were compared using linear mixed models. Graft survival was estimated using the Kaplan–Meier method and compared using the log-rank test. Cox proportional hazards model analyses were used to predict graft survival. The multivariate analysis was performed using the factors from the univariate analysis that were statistically significant (*p* < 0.05) and clinically significant factors that were not statistically significant in the univariate analysis. All tests were two-tailed, and statistical significance was defined as *p* < 0.05. All statistical analyses were done using SPSS version 25.0 (SPSS, Inc., IBM Corporation, Armonk, NY, USA).

## Supplementary Information

Supplementary Information 1.

Supplementary Information 2.

## Abbreviations

ACR: Acute cellular rejection
AKI: Acute kidney injury
AMR: Antibody-mediated rejection
BMI: Body mass index
BPAR: Biopsy-proven acute rejection
DBD: Donation after brain death
DGF: Delayed graft function
DM: Diabetes mellitus
ECD: Expanded criteria donor
eGFR: Estimated glomerular filtration rate
ESRD: End-stage renal disease
HCV: Hepatitis c virus
HLA: Human leukocyte antigen
HTN: Hypertension
KDIGO: Kidney Disease: Improving Global Outcomes
KDPI: Kidney donor profile index
KDRI: Kidney donor risk index
KONOS: Korea network for organ sharing
KT: Kidney transplantation
MMF: Mycophenolate mofetil
MPD: Methylprednisolone
r-ATG: Rabbit anti-thymocyte globulin
SCD: Standard criteria donor
SCr: Serum creatinine
