# Supplementary material for: Safety and effectiveness of kidney transplantation using a donation after brain death donor with acute kidney injury: a retrospective cohort study
Source: Sci Rep. 2021 Mar 10;11:5572. doi: 10.1038/s41598-021-84977-1 (PMC7946918; doi:10.1038/s41598-021-84977-1)
Supplement: Supplementary file 1 — Supplementary Information 1. [file 41598_2021_84977_MOESM1_ESM.docx]

**Safety and effectiveness of kidney transplantation using a donation after brain death donor with acute kidney injury: A retrospective cohort study**

Kyeong Deok Kim, MD^1^, Kyo Won Lee, MD^1^*, Sang Jin Kim, MD^1^, Okjoo Lee, MD^1^, Manuel Lim, MD^1^, Eun Sung Jeong, MD^1^, Jieun Kwon, MD^1^, Jaehun Yang, MD^1^, Jongwook Oh, MD^2^ & Jae Berm Park, MD^1^

^1^ Department of Surgery, Samsung Medical Center, Sungkyunkwan University School of Medicine, Seoul, Republic of Korea.

^2^ Department of Surgery, Samsung Changwon Hospital, Sungkyunkwan University School of Medicine, Changwon, Republic of Korea.

**ORCID:**

Kyeong Deok Kim https://orcid.org/0000-0002-4407-2909

Kyo Won Lee https://orcid.org/0000-0002-2722-7817

**Correspondence to:**

Kyo Won Lee, MD

Department of Surgery, Samsung Medical Center, Sungkyunkwan University School of Medicine, Seoul 06355, Republic of Korea.

Tel: 82-02-3410-0842; Fax: 82-02-3410-0040; E-mail: kw1980.lee@gmail.com

| **SUPPLEMENTARY TABLE S1.**  Clinical characteristics of donor with AKI according to induction agents | | | | | |
| --- | --- | --- | --- | --- | --- |
|  | | Basiliximab (n = 110) | High dose r-ATG (n = 59) | Low dose r-ATG (n = 90) | *P-*value |
| Age (years) | | 45.8 ± 13.8 | 44.7 ± 13.4 | 52.7 ± 13.8 | <0.001 |
| Sex (n, % male) | | 79 (71.8) | 42 (71.2) | 55 (61.1) | 0.226 |
| BMI (kg/m^2^) | | 23.0 ± 4.0 | 23.6 ± 2.6 | 24.6 ± 2.9 | 0.006 |
| Cormobidities | |  |  |  |  |
|  | Diabetes mellitus (n, %) | 7 (6.5) | 7 (12.3) | 17 (19.8) | 0.022 |
|  | Hypertension (n, %) | 23 (21.7) | 15 (26.3) | 27 (31.4) | 0.314 |
| HCV (n, %) | | 0 (0) | 2 (3.4) | 0 (0) | 0.052 |
| Cause of death (n, %) | |  |  |  | 0.005 |
|  | Cerebrovascular accident | 57 (51.8) | 31 (52.5) | 42 (46.7) |  |
|  | Trauma | 24 (21.8) | 17 (28.8) | 16 (17.8) |  |
|  | Hypoxic brain damage | 21 (19.1) | 7 (11.9) | 32 (35.6) |  |
|  | Other | 8 (7.3) | 4 (6.8) | 0 (0) |  |
| Donor's status (ECD) (n, %) | | 24 (21.8) | 14 (23.7) | 45 (50.0) | <0.001 |
| Kidney Donor Risk Index | | 1.14 ± 0.35 | 1.19 ± 0.36 | 1.43 ± 0.56 | <0.001 |
| Kidney Donor Profile Index | | 57.6 ± 23.7 | 60.9 ± 22.7 | 71.0 ± 22.9 | <0.001 |
| Creatinine level (mg/dl) | |  |  |  |  |
|  | Initial | 1.33 ± 0.76 | 2.07 ± 1.74 | 1.26 ± 0.92 | 0.005 |
|  | Peak | 1.94 ± 0.82 | 3.10 ± 1.91 | 3.08 ± 1.69 | <0.001 |
|  | Terminal | 1.45 ± 0.76 | 2.31 ± 1.09 | 2.48 ± 1.45 | <0.001 |
| Cold ischemia time (min) | | 286.1 ± 137.5 | 302.2 ± 187.5 | 264.8 ± 129.7 | 0.240 |
| Follow up duration (years) | | 7.1 [4.9-10.1] | 9.2 [5.9-11.6] | 5.0 [4.0-6.6] | <0.001 |

Continuous variables given as mean ± SD or as median [P25-P75].

ATG, anti-thymocyte globulin; BMI, body mass index; HCV, hepatitis c virus; ECD, expanded criteria donor.

| **SUPPLEMENTARY TABLE S2.**  Patient survival and death-censored graft survival rates. | | | | | |
| --- | --- | --- | --- | --- | --- |
|  | No AKI (n = 117) | AKI (n = 259) | KDIGO stage 1 (n = 102) | KDIGO stage 2 (n = 71) | KDIGO stage 3 (n = 86) |
| 2-year PS and DCGS rate (% / %) | 98.3 / 96.5 | 92.2 / 95.2 | 90.0 / 91.9 | 95.8 / 98.5 | 91.8 / 96.5 |
| 4-year PS and DCGS rate (% / %) | 96.4 / 91.1 | 91.0 / 93.5 | 86.6 / 90.8 | 94.3 / 93.9 | 91.8 / 96.5 |
| 6-year PS and DCGS rate (% / %) | 96.4 / 87.1 | 89.9 / 88.7 | 85.0 / 80.5 | 94.3 / 92.0 | 91.8 / 95.1 |
| 8-year PS and DCGS rate (% / %) | 92.4 / 81.0 | 87.6 / 78.6 | 85.0 / 68.8 | 89.5 / 79.0 | 89.4 / 89.8 |
| 10-year PS and DCGS rate (% / %) | 92.4 / 75.1 | 86.8 / 76.1 | 78.5 / 68.8 | 83.9 / 74.1 | 89.4 / 86.2 |

PS, patient survival; DCGS, death-censored graft survival.
