# Supplementary figures and images for: Safety and effectiveness of kidney transplantation using a donation after brain death donor with acute kidney injury: a retrospective cohort study
Source: Sci Rep. 2021 Mar 10;11:5572. doi: 10.1038/s41598-021-84977-1 (PMC7946918; doi:10.1038/s41598-021-84977-1)

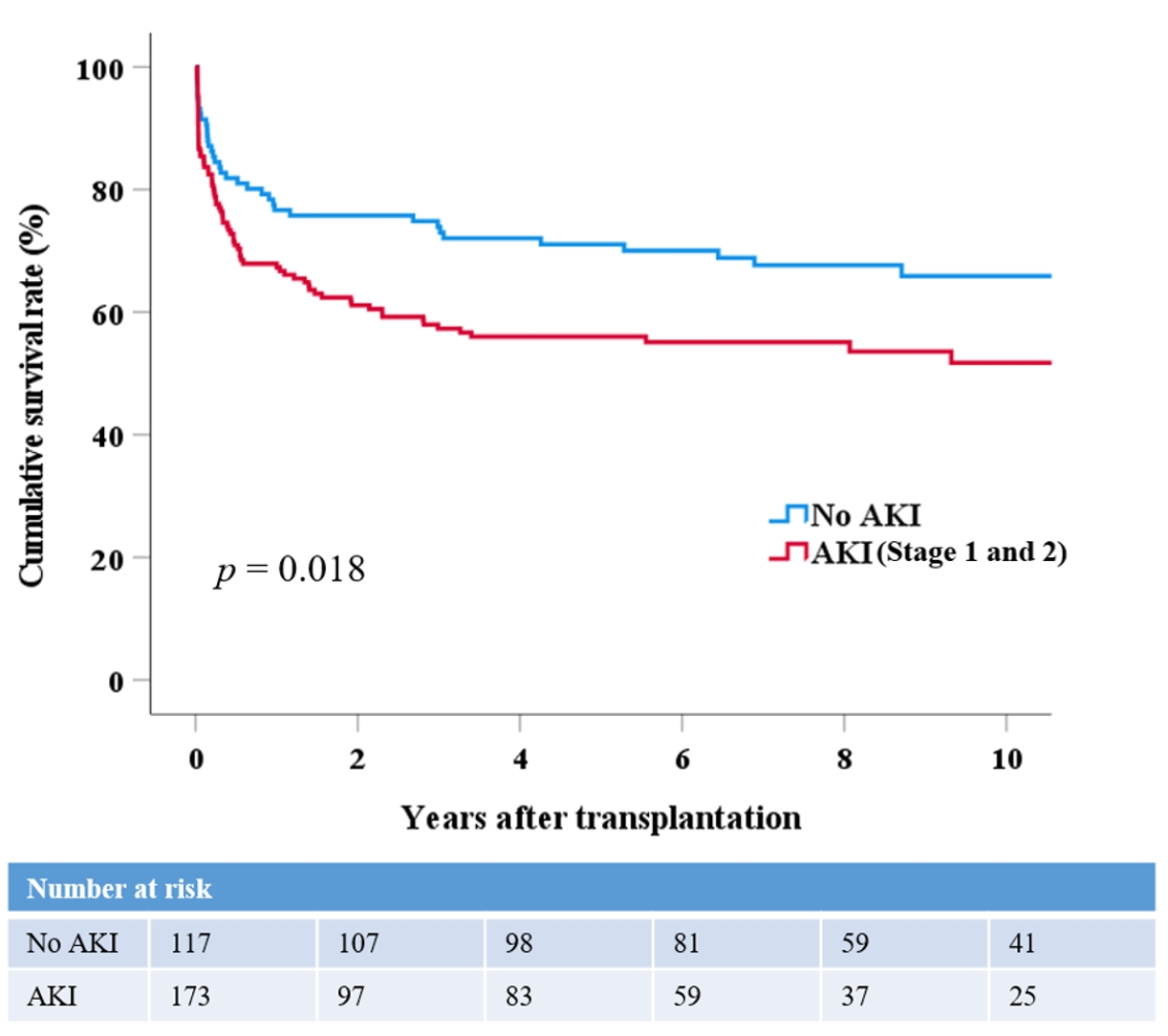

Supplement: Supplementary file 2 — Supplementary Information 2. [file 41598_2021_84977_MOESM2_ESM.tif]
